# Exosomal miR-19a from adipose-derived stem cells suppresses differentiation of corneal keratocytes into myofibroblasts

**DOI:** 10.18632/aging.102802

**Published:** 2020-02-29

**Authors:** Ting Shen, Qingqing Zheng, Hongbo Luo, Xin Li, Zhuo Chen, Zeyu Song, Guanfang Zhou, Chaoyang Hong

**Affiliations:** 1 Department of Ophthalmology, Zhejiang Provincial People’s Hospital and People’s Hospital of Hangzhou Medical College, Hangzhou 310014, Zhejiang, P. R. China; 2 Wenzhou School of Ophthalmology and Optometry, Wenzhou Medical University, Wenzhou 325035, Zhejiang, P. R. China; 3 Bengbu Medical College, Bengbu 233030, Anhui, P. R. China; 4 Department of Ophthalmology, Zhejiang Hospital, Hangzhou 310007, Zhejiang, P. R. China

**Keywords:** corneal stroma, keratocytes, adipose-derived stem cells, exosomes, miRNA

## Abstract

In this study, we investigated the effects of exosomal microRNAs (miRNAs) from adipose-derived stem cells (ADSCs) on the differentiation of rabbit corneal keratocytes. Keratocytes grown in 10% FBS differentiated into myofibroblasts by increasing HIPK2 kinase levels and activity. HIPK2 enhanced p53 and Smad3 pathways in FBS-induced keratocytes. Keratocytes grown in 10% FBS also showed increased levels of pro-fibrotic proteins, including collagen III, MMP9, fibronectin, and α-SMA. These effects were reversed by knocking down HIPK2. Moreover, ADSCs and exosomes derived from ADSCs (ADSCs-Exo) suppressed FBS-induced differentiation of keratocytes into myofibroblasts by inhibiting HIPK2. Quantitative RT-PCR analysis showed that ADSCs-Exos were significantly enriched in miRNA-19a as compared to ADSCs. Targetscan and dual luciferase reporter assays confirmed that the HIPK2 3’UTR is a direct binding target of miR-19a. Keratocytes treated with 10% FBS and ADSCs-Exo-miR-19a-agomir or ADSCs-Exo-NC-antagomir showed significantly lower levels of HIPK2, phospho-Smad3, phospho-p53, collagen III, MMP9, fibronectin and α-SMA than those treated with 10% FBS plus ADSCs-Exo-NC-agomir or ADSCs-Exo-miR-19a-antagomir. Thus, exosomal miR-19a derived from the ADSCs suppresses FBS-induced differentiation of rabbit corneal keratocytes into myofibroblasts by inhibiting HIPK2 expression. This suggests their potential use in the treatment of corneal fibrosis.

## INTRODUCTION

The cornea is the outermost part of the eye that acts as a barrier against infections and provides a clear path for light [1]. The cornea is made up of three layers: epithelium, stroma, and endothelium [2]. Nearly 90% of the corneal volume is made up of the stroma, which is primarily responsible for clarity and ocular immunity [3]. The corneal stromal tissue is primarily made up of collagen fibers and extracellular matrix [4]. Keratocytes are the major cells of the stroma that produce collagen and matrix metalloproteinases [5]. However, wound healing response to corneal injury, infections, and surgery decrease corneal transparency and visual acuity [6]. During stromal wound healing, keratocytes differentiate into fibroblasts and myofibroblasts [7]. Moreover, deposition of extracellular matrix and decreased crystallin protein expression by the keratocytes causes scar formation and reduces corneal transparency [8]. Therefore, effective methods are necessary to inhibit differentiation of keratocytes into myofibroblasts in response to injury and restore corneal stroma.

Adipose-derived stem cells (ADSCs) are a type of adult stem cells isolated easily from the human adipose tissues with an ability to self-renew and differentiate into endothelium, bone, muscle, fat, and cartilage tissue types [9, 10]. Moreover, ADSCs can be *in situ* differentiated into functional keratocytes, *and* are safe and non-immunogenic [11]. Arnalich-Montiel et al demonstrated that ADSCs are a potential source of stem cell therapy for damaged corneas [2].

Cao et al demonstrated *in vitro* differentiation of ADSCs into myocytes, and low rate of myogenesis when ADSCs were injected into gastrocnemius muscle of mdx mice [12]. ADSCs repair tissue damage by secreting paracrine factors and exosomes [12, 13]. The exosomes are extracellular vesicles of approximately 40–100 nm in size that are generated by several cells and tissues [14, 15]. Exosomes derived from ADSCs (ADSCs-Exo) evade the immune rejection responses of the host and accelerate wound healing by inducing the migration of fibroblasts [16, 17]. Our previous study found that ADSCs restore corneal stroma and remodel the ECM by secreting exosomes [18]. Hu et al showed that ADSCs-Exo promotes cutaneous wound healing by inhibiting collagen expression and reducing scar formation [19]. Transmembrane proteins such as CD9, CD63, and CD81 are highly enriched on the exosomal membranes [20]. ADCSs-Exo act as key mediators of intercellular communication and deliver proteins, lipids, miRNAs, and mRNAs to the recipient cells [17, 21]. Fen et al demonstrated that angiogenesis was stimulated by miR-423-5p transferred into the umbilical vein endothelial cells via exosomes [22]. However, the mechanism by which ADSCs-Exo reduces scar formation and regulates corneal stromal repair remains to be elucidated.

Homeodomain-interacting protein kinase 2 (HIPK2) is a serine/threonine kinase that is primarily located in the nucleus of eukaryotic cells [23]. HIPK2 is a pro-fibrotic gene that plays an important role in kidney fibrosis [24]. Previous studies show that HIPK2 regulates fibrosis by acting upstream of p53, Transforming Growth Factor β (TGF-β), SMAD family member 3 (Smad3), and INT-1 (Wnt)/β-catenin pathways [24, 25]. Hu et al showed that exosomal miR-1229 promotes angiogenesis of colorectal cancer cells by targeting HIPK2 [26]. The relationship between exosome-derived miRNAs secreted by ADSCs and HIPK2 is not known. Therefore, the aim of this study was to investigate if the exosome-derived miRNAs secreted by ADSCs regulated differentiation of keratocytes into myofibroblasts using the rabbit corneal keratocytes and ADSCs.

## RESULTS

### FBS induces differentiation of rabbit corneal keratocytes into myofibroblasts

**Figure 1:**
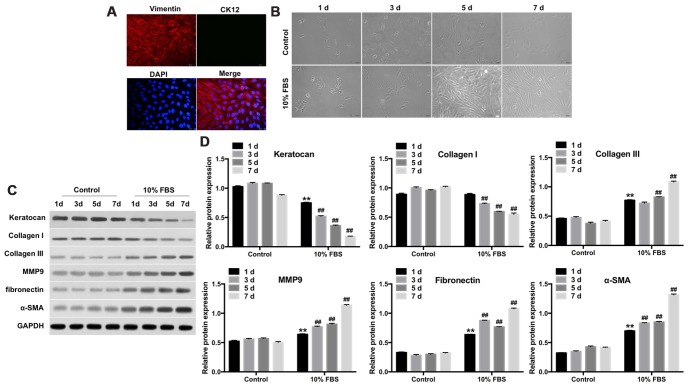
**FBS induces differentiation of rabbit corneal keratocytes into myofibroblasts.** (**A**) Representative immunofluorescence images show staining of rabbit corneal stromal cells with fluorescent-tagged antibodies against Vimentin (red) and CK12 (green). Vimentin-positive cells are the keratocytes; CK12-positive cells are the corneal epithelial cells; Diamidinophenylindole (DAPI; blue) stains the nucleus; magnification: 200x. (**B**) Representative phase-contrast images show phenotypic features of rabbit corneal keratocytes cultured in 10% FBS or serum-free DMEM/F12 medium for 1, 3, 5 and 7 days. The cell cultures were observed under a light microscope and images were captured at 10X magnification. (**C**) Representative western blot images show levels of keratocan, collagen I, collagen III, MMP9, fibronectin and α-SMA proteins in rabbit keratocyte cells grown in DMEM/F12 medium containing 10% FBS or serum-free medium for 7 days. GAPDH was used as an internal control. (**D**) Histogram plot shows keratocan, collagen I, collagen III, MMP9, fibronectin and α-SMA protein levels relative to GAPDH. ** denotes P < 0.01 compared with the control (1d) group; ^##^ denotes P < 0.01 compared with the 10% FBS (1d) group.

Previous studies have reported that vimentin and CK12 are specifically expressed in the keratocytes and corneal epithelium, respectively [27, 28]. Therefore, we examined the expression of stromal and epithelial markers in primary rabbit keratocytes by immunofluorescence assay. The primary rabbit keratocytes showed positive expression of vimentin, but did not express CK12 (Figure 1A). This confirmed that the rabbit corneal stroma cells were keratocytes and not epithelial cells.

Keratocytes can be differentiated into myofibroblasts when grown in presence of FBS [29]. We observed that keratocytes grown in serum-free medium showed dendritic morphology, whereas, keratocytes cultured with 10% FBS for 7 days exhibited a fibroblast phenotype (Figure 1B). Furthermore, we performed western blot analysis of the expression of fibroblast-related proteins, such as, keratocan, collagen I, collagen III, MMP9, fibronectin and α-SMA in the cultured keratocytes. Keratocytes grown in 10% FBS showed significantly reduced expression of keratocan and collagen I and increased levels of collagen III, MMP9, fibronectin and α-SMA compared to the controls (Figure 1C, 1D). These data demonstrate that FBS induced differentiation of keratocytes into myofibroblasts.

### Characterization of ADSCs and ADSCs-Exo

**Figure 2:**
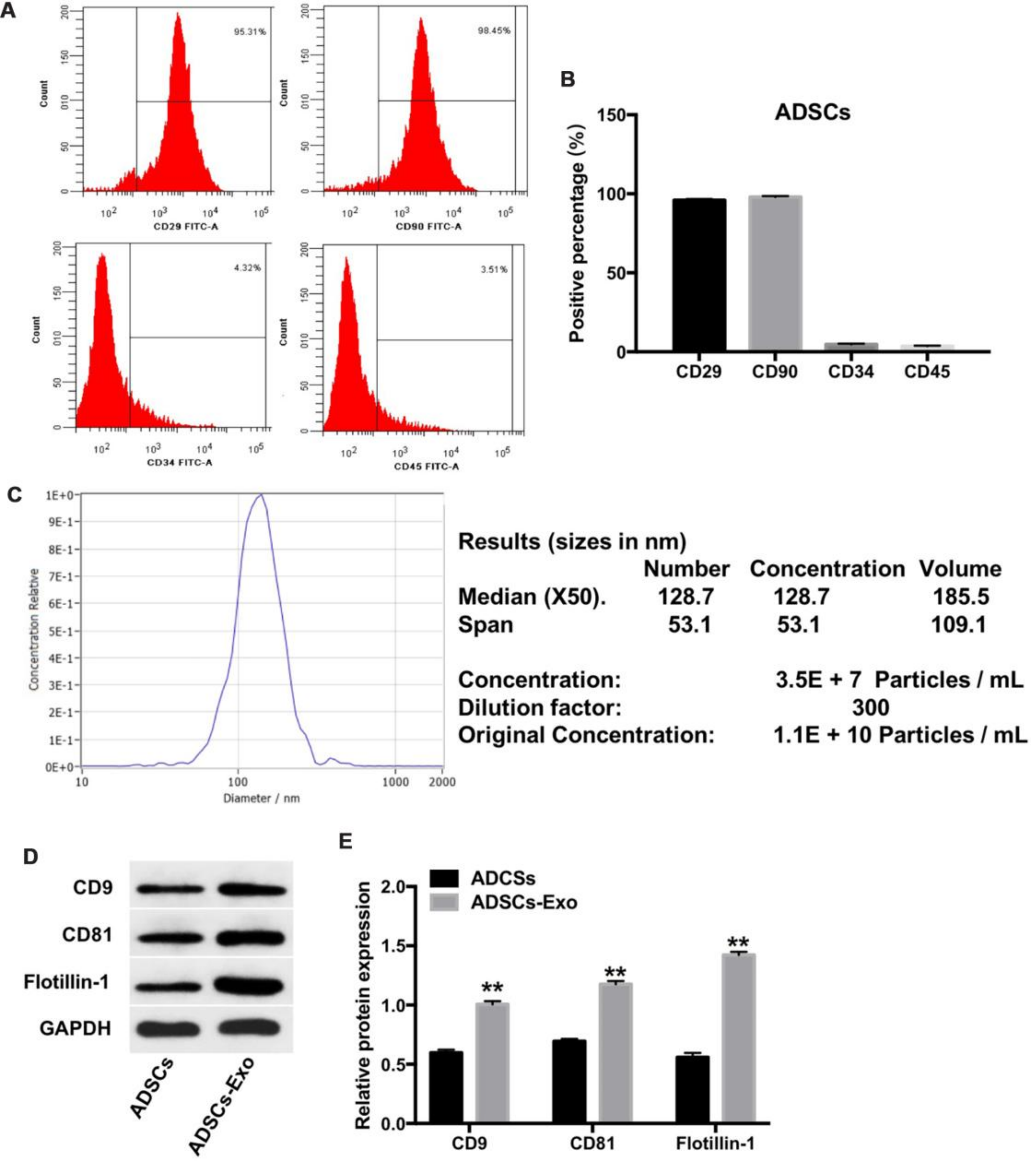
**Characterization of ADSCs and ADSCs-Exo.** (**A**, **B**) Flow cytometry analysis of ADSCs isolated from rabbit adipose tissues is shown using fluorescent-tagged antibodies against cell surface proteins, namely, CD29, CD90, CD34, and CD45. (**C**) The mean diameter of ADSCs exosomes was analyzed using a nanoparticle tracking system (NTA). NTA analysis of the exosomes isolated from ADSCs (ADSCs-Exo) shows a mean concentration of 1.1 x 10^10^ particles per mL. (**D**, **E**) Western blot analysis shows levels of CD9, CD81 and flotillin-1 proteins in the ADSCs and the ADSCs-Exo. GAPDH was used as an internal control. The levels of CD9, CD81 and flotillin-1 are expressed relative to GAPDH. ** denotes P < 0.01 compared with the ADSC group.

Next, we characterized the ADSCs isolated from rabbit adipose tissues. Flow cytometry analysis showed positive surface expression of CD29 and CD90 and absence of CD34 and CD45 expression in the primary ADSCs (Figure 2A, 2B). This confirmed successful isolation of ADSCs from the rabbit adipose tissues.

Furthermore, we analyzed the exosomes isolated from the ADSCs. Nanoparticle tracking analysis (NTA) showed that the ADSCs-Exo were approximately 100 nm in diameter with typical cup-shaped morphology (Figure 2C). Western blot analysis showed higher expression of exosomal markers, namely, CD9, CD81 and flotillin-1 in the ADSCs-Exo compared with the ADSCs (Figure 2D, 2E). These data confirmed isolation of purified ADSCs-Exo from the ADSC culture supernatants.

### ADSCs-Exo inhibits FBS-induced differentiation of keratocytes into myofibroblasts

**Figure 3:**
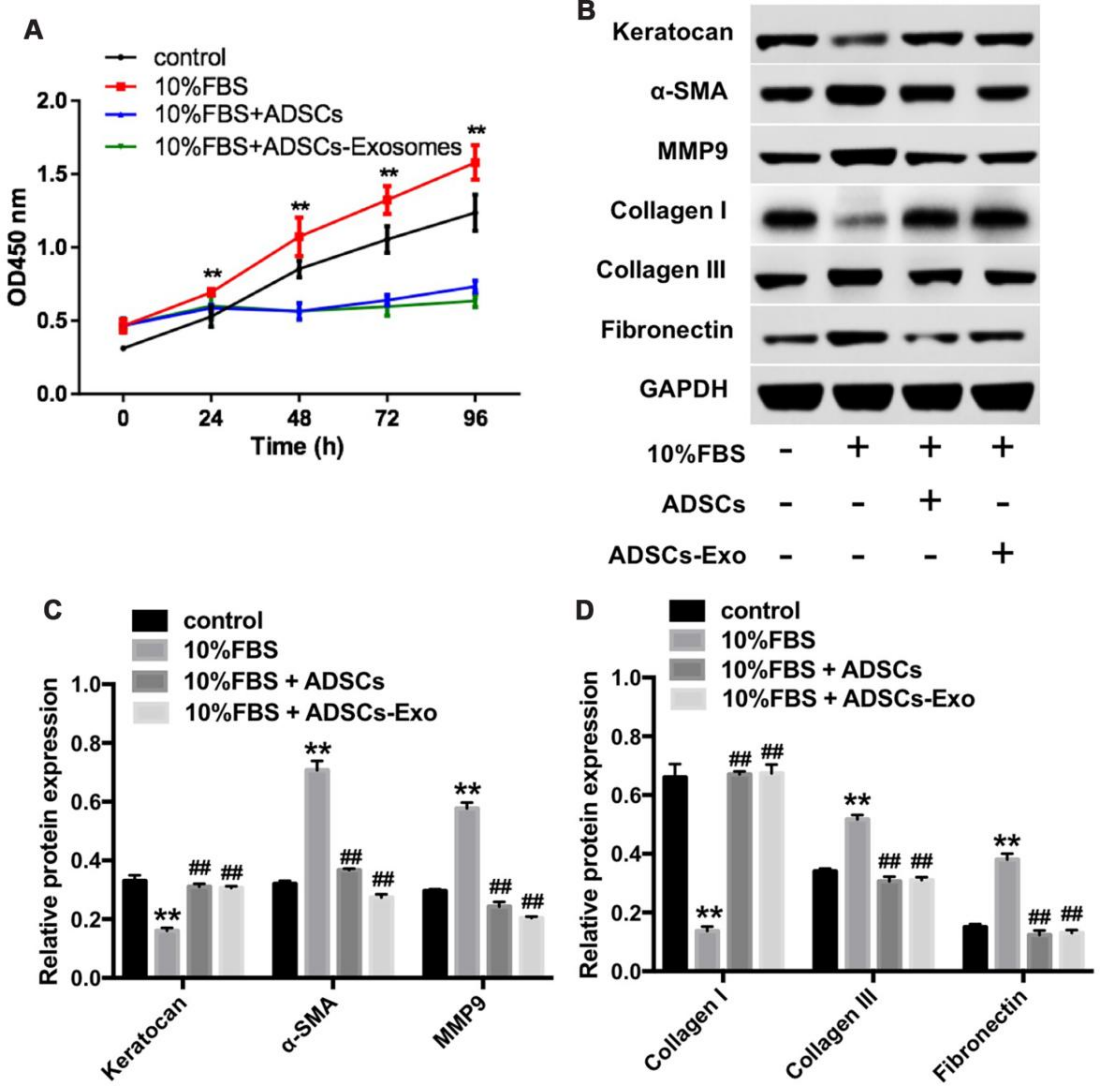
**ADSCs-Exo inhibits FBS-induced differentiation of keratocytes into myofibroblasts.** (**A**) CCK-8 assay results show the viability of rabbit corneal keratocytes incubated with ADSCs or ADSCs-Exo in the presence of 10% FBS for 0, 24, 48, 72 and 96 h. (**B**) Representative western blotting images show levels of keratocan, α-SMA, MMP9, collagen I, collagen III, and fibronectin proteins in rabbit corneal keratocytes incubated with ADSCs or ADSCs-Exo in the presence of 10% FBS for 96 h. GAPDH was used as an internal control. (**C**, **D**) Histogram plots show the levels of keratocan, α-SMA, MMP9, collagen I, collagen III, and fibronectin proteins relative to GAPDH in rabbit corneal keratocytes incubated with ADSCs or ADSCs-Exo in the presence of 10% FBS for 96 h, as determined by western blotting. ** denotes P < 0.01 as compared with the control group; ^##^ denotes P < 0.01as compared with the 10% FBS group.

We then characterized the effects of ADSCs-Exo on the keratocytes that were grown in DMEM/F12 medium containing 10% FBS for 7 days. CCK-8 cell proliferation assay showed that 10% FBS significantly induced proliferation of corneal myofibroblasts at 24, 48, 72, and 96 h time points, whereas, FBS-induced keratocyte cell proliferation was significantly inhibited by ADSCs and ADSCs-Exo at 48, 72 and 96 h (Figure 3A). Moreover, keratocytes grown in DMEM/F12 medium containing 10% FBS showed significantly reduced keratocan and collagen I protein expression, and increased collagen III, MMP9, fibronectin and α-SMA protein levels compared with those grown in serum-free medium, but, these FBS-induced changes were inhibited when keratocytes were grown in presence of ADSCs or ADSCs-Exo (Figure 3B–3D). These data suggest that ADSCs and ADSCs-Exo inhibit differentiation of keratocytes into myofibroblasts and the proliferation of myofibroblasts.

### HIPK2 downregulation suppresses FBS-induced differentiation of keratocytes into myofibroblasts by inhibiting p53 and Smad3 signaling pathways

**Figure 4:**
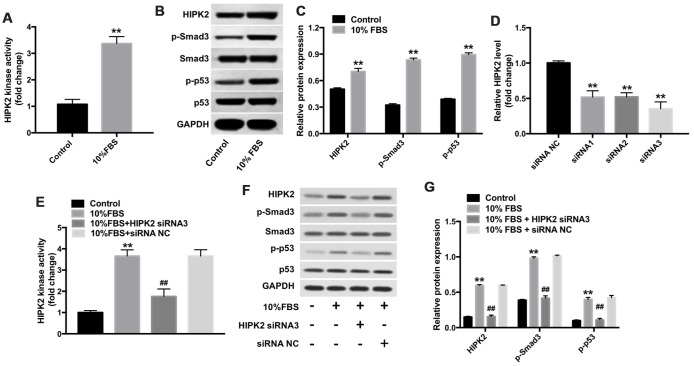
**Downregulation of HIPK2 suppresses FBS-induced differentiation of rabbit corneal keratocytes into myofibroblasts.** (**A**) Histogram plot shows HIPK2 kinase activity in rabbit corneal keratocytes grown in DMEM/F12 medium with and without 10% FBS. (**B**, **C**) Western blot analysis shows levels of HIPK2, Smad3, p-Smad3, p53, and p-p53 in rabbit corneal keratocytes grown in DMEM/F12 medium with and without 10% FBS. GAPDH was used as internal control. Histogram plot shows levels of HIPK2, p-Smad3, and p-p53 relative to GAPDH, Smad3, and p53, respectively. (**D**) QRT-PCR analysis shows relative HIPK2 mRNA levels in rabbit corneal keratocytes transfected with NC-siRNA, HIPK2-siRNA1, HIPK2-siRNA2, and HIPK2-siRNA3 for 48 h. β-actin mRNA levels were used for normalization. (**E**) HIPK2 kinase activity in rabbit corneal keratocytes, transfected with NC-siRNA or HIPK2-siRNA3, and grown in DMEM/F12 medium with 10% FBS for 48 h. (**F**, **G**) Western blot analysis shows HIPK2, Smad3, p-Smad3, p53, and p-p53 levels in rabbit corneal keratocytes, transfected with NC-siRNA or HIPK2-siRNA3, and grown in DMEM/F12 medium with 10% FBS for 48 h. GAPDH was used as internal control. Histogram plot shows levels of HIPK2, p-Smad3, and p-p53 relative to GAPDH, Smad3, and p53, respectively. ** denotes P < 0.01 as compared with the control group; ^##^ denotes P < 0.01 as compared with the 10% FBS + NC-siRNA group.

HIPK2 is a pro-fibrotic gene that modulates p53 and TGF-β/Smad3 signaling pathways [24, 25]. HIPK2 kinase activity is significantly increased in keratocytes cultured with 10% FBS for 7 days compared to controls (Figure 4A). Western blot analysis showed increased expression of HIPK2, p-Smad3 and p-p53 in keratocytes grown in medium containing 10% FBS compared with the control group (Figure 4C).

Next, we used three different siRNAs (HIPK2-siRNA1, HIPK2-siRNA2 and HIPK2-siRNA3) to knock down HIPK2 in the keratocytes. Keratocytes transfected with HIPK2-siRNA3 showed significant downregulation of HIPK2 when cultured in medium containing 10% FBS compared with NC-siRNA transfected keratocytes (Figure 4D). Moreover, HIPK2-knockdown keratocytes showed significantly reduced 10% FBS-induced HIPK2 activity compared with the controls (Figure 4E). Furthermore, HIPK2, p-Smad3 and p-p53 protein levels were significantly reduced in HIPK2-knockdown corneal keratocytes compared with controls, when grown in medium containing 10% FBS (Figure 4F and 4G). Furthermore, downregulation of HIPK2 inhibited the myofibroblast phenotype promoted by FBS (Supplementary Figure 1A). These data suggest that downregulation of HIPK2 suppresses differentiation of keratocytes into myofibroblasts by inhibiting the p53 and Smad3 signaling pathways.

### HIPK2 is the direct binding target of miR-19a

**Figure 5:**
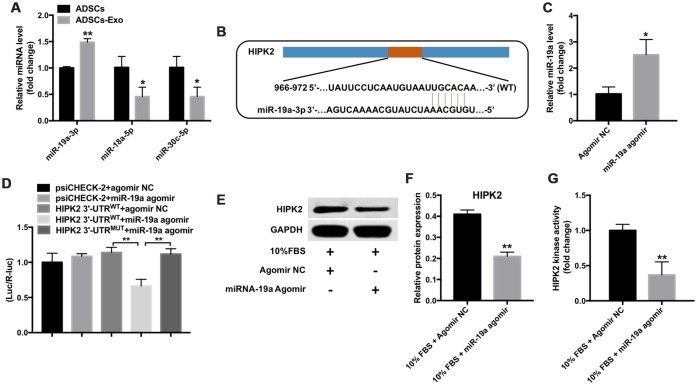
**Mir-19a directly binds to the HIPK2-3’UTR.** (**A**) QRT-PCR analysis shows miR-19a, miR-18a-5p and miR-30c-5p levels in ADSCs and ADSCs-Exo. ** denotes P < 0.01 in comparison with the ADSC group. (**B**) Targetscan analysis shows the sequence, 5’-UGCACA-3’, as the predicted target site of miR-19a in the 3’UTR region of the *HIPK2* gene between 966-972 nucleotides. (**C**) QRT-PCR analysis shows levels of miR-19a in the rabbit corneal keratocytes transfected with miR-19a-agomir or NC-agomir for 48 h. ** denotes P < 0.01 in comparison with the agomir NC group. (**D**) The dual luciferase reporter assay shows relative luciferase activity in the 293T cells co-tranfected with the plasmids containing HIPK2-WT-3’UTR or HIPK2-MUT-3’UTR and miR-19a agomir. (**E**, **F**) Western blotting analysis shows HIPK2 protein expression in rabbit keratocytes, grown in DMEM/F12 medium with 10% FBS, and transfected with miR-19a-agomir or NC-agomir for 48 h. Histogram plot shows HIPK2 protein levels relative to GAPDH. ** denotes P < 0.01 when compared with the 10% FBS + NC-agomir group. (**G**) Histogram plot shows HIPK2 kinase activity in rabbit keratocytes grown in DMEM/F12 medium with 10% FBS and transfected with miR-19a agomir or NC-agomir for 48 h. ** denotes P < 0.01 as compared with the 10% FBS + NC-agomir group.

Next, we investigated the effects of ADSCs-Exo on the differentiation of keratocytes into myofibroblasts. Exosomes act as key mediators of intercellular communication by delivering miRNAs such as miR-19a-3p, miR-18a-5p and miR-30c-5p to recipient cells [30, 31]. QRT-PCR analysis showed that miR-19a-3p levels were significantly higher in the ADSCs-Exo compared with the ADSCs (Figure 5A).

TargetScan analysis suggested that HIPK2 is a potential target of miR-19a-3p (Figure 5B). Moreover, miR-19a levels were significantly upregulated in miR-19a agomir transfected keratocytes cultured in medium containing 10% FBS compared with the controls (Figure 5C). Dual luciferase reporter assay results showed that miR-19a suppressed the luciferase activity of the psiCHECK-2-HIPK2-WT construct, but did not affect the luciferase activity of the psiCHECK-2-HIPK2-MUT construct (Figure 5D). These results confirmed that miR-19a directly targeted the 3’-UTR of HIPK2.

Furthermore, miR-19a-agomir transfected keratocytes showed significantly decreased HIPK2 protein levels and activity compared to NC-agomir transfected keratocytes (Figure 5E–5G). These data suggest that overexpression of miR-19a decreases HIPK2 levels and activity in keratocytes cultured with 10% FBS.

### Exosomal miR-19a derived from the ADSCs inhibits FBS-induced differentiation of keratocytes into myofibroblasts by suppressing HIPK2 expression

**Figure 6:**
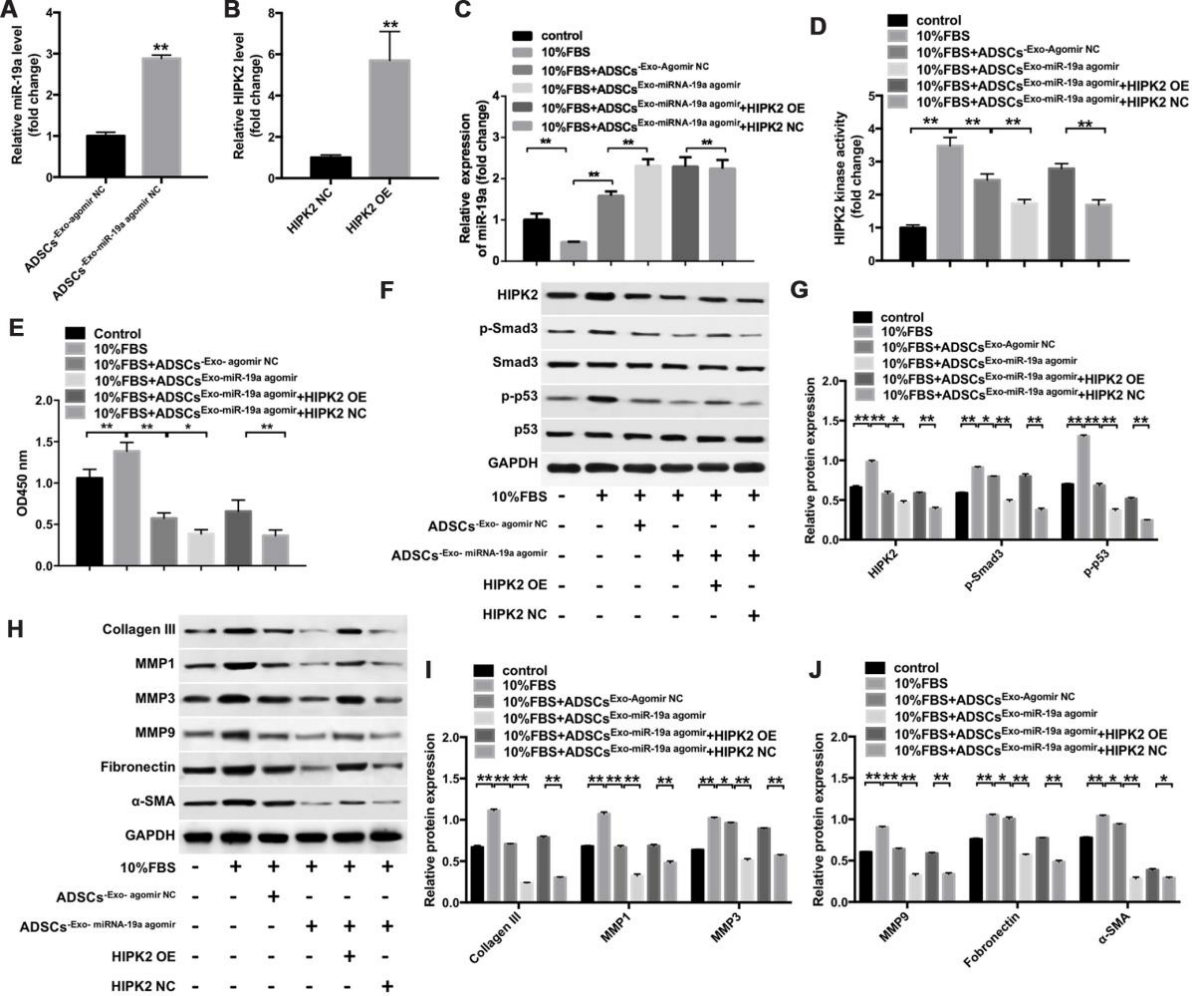
**ADSCs-Exo-miR-19a suppresses FBS-induced differentiation of rabbit corneal keratocytes into myofibroblasts by inhibiting HIPK2 expression.** (**A**) QRT-PCR analysis shows miR-19a levels in ADSC-Exo obtained from ADSCs that were transfected with miR-19a-agomir (ADSCs-Exo-miR-19a-agomir) or NC-agomir (ADSCs-Exo-NC-agomir) for 48 h. ** denotes P < 0.01 as compared with the ADSCs-Exo-NC-agomir group. (**B**) QRT-PCR analysis shows HIPK2 mRNA levels in rabbit corneal keratocytes, transfected with lenti-NC or lenti-HIPK2 for 48 h. β-actin was used as internal control. ** denotes P < 0.01 as compared with the HIPK2-NC group. (**C**) QRT-PCR analysis shows miR-19a levels in lenti-HIPK2 transfected rabbit corneal keratocytes, grown in DMEM/F12 medium with 10% FBS, and incubated with 100 μg/mL ADSCs-Exo-miR19a-agomir or 100 μg/mL ADSCs-Exo-miR19a-agomir. Exosomes were obtained by ultracentrifugation of the cell culture supernatant of ADSCs that were transfected with miR-19a-agomir or NC-agomir for 48 h. ** denotes P < 0.01. (**D**) Histogram plot shows HIPK2 kinase activity in lenti-HIPK2 transfected rabbit corneal keratocytes, grown in DMEM/F12 medium with 10% FBS, and incubated with 100 μg/mL ADSCs-Exo-miR19a-agomir or 100 μg/mL ADSCs-Exo-NC-agomir. ** denotes P < 0.01. (**E**) CCK-8 assay results show viability of lenti-HIPK2 transfected rabbit corneal keratocytes, grown in DMEM/F12 medium with 10% FBS, and incubated with 100 μg/mL ADSCs-Exo-miR19a-agomir or 100 μg/mL ADSCs-Exo-NC-agomir at 0, 2, 48, and 72 h. (**F**, **G**) Western blot analysis shows HIPK2, Smad3, p-Smad3, p53, and p-p53 in lenti-HIPK2 transfected rabbit corneal keratocytes, grown in DMEM/F12 medium with 10% FBS, and incubated with 100 μg/mL ADSCs-Exo-miR19a-agomir or 100 μg/mL ADSCs-Exo-NC-agomir. The levels of HIPK2, p-Smad3, and p-p53 proteins are expressed relative to GAPDH, Smad3, and p53, respectively. (**H**) Western blot analysis of the levels of collagen III, MMP1, MMP3, MMP9, fibronectin and α-SMA proteins in lenti-HIPK2 transfected rabbit corneal keratocytes, grown in DMEM/F12 medium with 10% FBS, and incubated with 100 μg/mL ADSCs-Exo-miR19a-agomir or 100 μg/mL ADSCs-Exo-NC-agomir. GAPDH was used as an internal control. (**I**, **J**) Histogram plots show the levels of (**I**) collagen III, MMP1, and MMP3 and (**J**) MMP9, fibronectin and α-SMA relative to GAPDH in lenti-HIPK2 transfected rabbit corneal keratocytes, grown in DMEM/F12 medium with 10% FBS, and incubated with 100 μg/mL ADSCs-Exo-miR19a-agomir or 100 μg/mL ADSCs-Exo-NC-agomir. ** denotes P < 0.01.

Next, we performed qRT-PCR assay to analyze if miR-19a derived from ADSCs-Exo affect differentiation of keratocytes into myofibroblasts. Keratocytes co-cultured with ADSCs-Exo showed significantly higher levels of miR-19a compared with the controls (Figure 6A). Moreover, lenti-HIPK2 transfected keratocytes showed significantly higher HIPK2 mRNA levels than the lenti-NC transfected keratocytes (Figure 6B). QRT-PCR analysis showed that miR-19a levels were significantly reduced in the keratocytes cultured with 10% FBS than the keratocytes cultured without FBS (Figure 6C). Furthermore, miR-19a levels were significantly higher in keratocytes cultured with 10% FBS and ADSCs-Exo-miR-19a-agomir compared with keratocytes cultured with 10% FBS and ADSCs-Exo-NC-agomir (Figure 6C). The HIPK2 kinase activity was significantly decreased in keratocytes cultured with 10% FBS ADSCs-Exo-miR-19a-agomir compared with keratocytes incubated with 10% FBS and ADSCs-Exo-NC-agomir, but, HIPK2 overexpression restored the HIPK2 kinase activity (Figure 6D). CCK8 assay showed that keratocytes incubated with ADSCs-Exo-miR-19a-agomir showed significantly reduced proliferation than the controls, but, HIPK2 overexpression increased the proliferation rate (Figure 6E). These data suggest that ADSCs-Exo-miR-19a inhibits proliferation of the keratocytes by targeting HIPK2.

**Figure 7:**
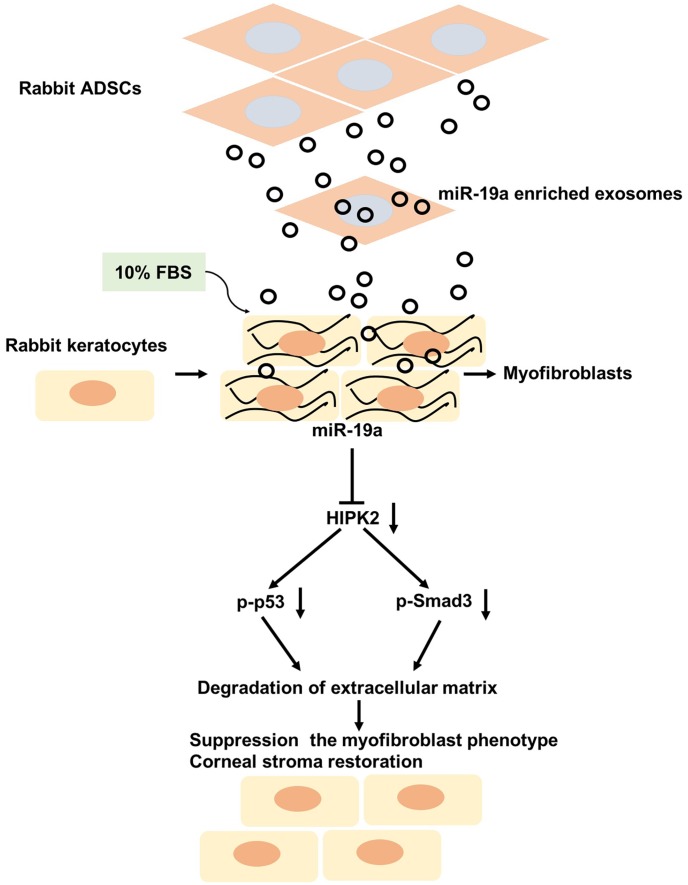
**Putative mechanism by which ADSCs-Exo-miR-19a suppress the FBS-induced differentiation of rabbit corneal keratocytes into myofibroblasts.** ADSCs-Exo-miR-19a suppresses FBS-induced differentiation of keratocytes into myofibroblasts by inhibiting HIPK2 expression. Reduced HIPK2 levels suppress the TGF-β/Smad3 and p53 signaling pathways, and reduce the expression of pro-fibrotic markers and ECM components. Overall, these events decrease cell viability and ECM degradation.

Next, we investigated if ADSCs-Exo-miR-19a agomir modulated the activity of the p53 and Smad3 signaling pathways when keratocytes were incubated with 10% FBS. Keratocytes cultured with 10% FBS and ADSCs-Exo showed significant reduction in HIPK2, p-Smad3 and p-p53 protein levels compared with the keratocytes incubated with 10% FBS alone (Figure 6F, 6G). Moreover, keratocytes incubated with 10% FBS and ADSCs-Exo-miR-19a agomir showed significantly reduced HIPK2, p-Smad3 and p-p53 protein levels compared with keratocytes incubated with 10% FBS and ADSCs-Exo-agomir NC; HIPK2 overexpression significantly increased HIPK2, p-Smad3 and p-p53 protein levels in keratocytes incubated with 10% FBS and ADSCs-Exo-miR-19a agomir (Figure 6F, 6G). Furthermore, keratocytes cultured with 10% FBS and ADSCs-Exo decreased collagen III, MMP1, MMP3, MMP9, fibronectin and α-SMA protein levels (Figure 6H–6J). Keratocytes cultured with 10% FBS and ADSCs-Exo-miR-19a agomir showed further reduction in the levels of collagen III, MMP1, MMP3, MMP9, fibronectin and α-SMA proteins, but, these levels were restored by HIPK2 overexpression using lenti-HIPK2 (Figure 6H–6J). Moreover, ADSCs-Exo-miR-19a inhibited the myofibroblast phenotype promoted by FBS, which was reversed following transfected with lenti-HIPK2 (Supplementary Figure 2A). These data indicate that ADSCs-Exo-miR-19a agomir suppresses differentiation of corneal keratocytes into myofibroblasts by inhibiting HIPK2 expression, and also decreases cell proliferation and ECM degradation (Figure 7). We also observed that keratocytes cultured with 10% FBS and ADSCs-Exo-miR-19a-antagomir showed significantly higher expression of HIPK2, Collagen III, Fibronectin and α-SMA proteins compared with keratocytes cultured with 10% FBS and ADSCs-Exo-NC-antagomir (Supplementary Figure 3A, 3B). Overall, these results suggest that exosomal miR-19a derived from the ADSCs inhibit FBS-induced differentiation of corneal keratocytes into myofibroblasts.

## DISCUSSION

Previous investigations show that corneal keratocytes incubated with 10% FBS differentiate into myofibroblasts [32]. Moreover, ADSCs can be induced to differentiate into functional keratocytes under specific growth conditions [1, 33]. ADSCs show immense therapeutic potential because they can mediate changes in cellular functions and signaling by secreting exosomes [34]. In this study, we demonstrate that ADSCs-Exo inhibit FBS-induced differentiation of corneal keratocytes by upregulating the levels of keratocan and collagen I, and downregulating the levels of collagen III, MMP9, fibronectin and α-smooth muscle action (α-SMA). Functional keratocytes express cornea-specific proteoglycans, such as keratocan and collagen I, but do not express collagen III [2, 35]. The myofibroblasts are characterized by high expressions of α-SMA, fibronectin and some ECM components [36]. Verhoekx et al showed that ADSCs inhibit myofibroblasts in Dupuytren's disease by downregulating α-SMA [37]. These data are consistent with our results, which show that ADSCs-Exo inhibit FBS-induced keratocyte differentiation, thereby suggesting the potential to regenerate the corneal stroma.

Exosomes are small vesicles that are released by all cells, and carry lipids, proteins, DNA, and RNAs, including mRNAs and miRNAs [38]. ADSCs-Exos are enriched with miRNAs that can modulate cellular functions in recipient cells [39]. Fang et al demonstrated that exosome-derived miRNA-21 and miR-23a suppress myofibroblast differentiation during wound healing by inhibiting the TGF-β/Smad signaling pathway [40]. Jin et al reported that the pro-fibrotic HIPK2 protein regulated fibrosis by modulating the activity of the pro-fibrotic TGF-β/Smad pathway and the pro-apoptotic p53 pathway [41]. Consistent with these reports, we observed that HIPK2, p-Smad3 and p-p53 levels were significantly upregulated in keratocytes cultured with 10% FBS, and reduced when HIPK2 levels were downregulated. Hence, our study suggests that HIPK2 is a key regulator of the p53 and Smad3 signaling pathways during the differentiation of keratocytes into myofibroblasts.

The results of the dual luciferase reporter assay confirmed that HIPK2 was a direct binding target of miR-19a. MiR-19a is enriched in the exosomes derived from the mesenchymal stromal cells [42]. In this study, we demonstrate that miR-19a is enriched in the exosomes derived from the ADSCs. Moreover, miR-19a in the ADSCs-Exo inhibits the expression of HIPK2 in the keratocytes cultured with 10% FBS. Souma et al showed that the miR-19a-19b-20a sub-cluster inhibits TGF-β-induced activation of fibroblasts in patients with pulmonary fibrosis [43]. Furthermore, miR-133b repairs corneal stroma by downregulating α-SMA and prevents scar formation [44]. Our data indicates that exosomal miR-19a derived from the ADSCs inhibits fibrosis by suppressing HIPK2.

HIPK2 activates TGF-β/Smad3 and p53 pathways, and promotes the expression of pro-fibrotic markers and ECM components, such as α-SMA, collagen III, MMP9, and fibronectin [24, 45]. We investigated if exosomal miR-19a inhibits differentiation of keratocytes into myofibroblasts via p53 and Smad3. ADSCs-Exo-miR-19a demonstrated anti-fibrotic activity by significantly reducing the levels of HIPK2, p-Smad3 and p-p53, which were induced by 10% FBS. Moreover, ADSCs-Exo-miR-19a decreased the levels of α-SMA and ECM-related proteins in keratocytes cultured with 10% FBS. This suggests that ADSCs-Exo-miR-19a can potentially regenerate corneal stroma by inhibiting keratocyte differentiation. Yin et al showed that ADSCs-Exo-miR-181-5p inhibits liver fibrosis by downregulating α-SMA [46]. Extracellular vesicles in the human serum contain miRNAs that inhibit liver fibrosis by decreasing the expression of pro-fibrotic genes [47]. The findings of our study are in agreement with these reports.

In conclusion, our study shows that ADSCs-Exo-miR-19a inhibits the differentiation of corneal keratocytes into myofibroblasts by suppressing HIPK2 expression. The downregulation of HIPK2 inhibits the TGF-β/Smad3 and p53 pathways, which results in reduced expression of pro-fibrotic markers and ECM components, thereby decreasing cell viability and ECM degradation. Therefore, our data indicates the therapeutic potential of ADSCs-Exo-miR-19a in repairing damaged corneal stromas.

## MATERIALS AND METHODS

### Isolation and culturing of primary rabbit corneal keratocytes

Ten week-old New Zealand male rabbits weighing 2.3 - 2.5 kg were purchased from the Zhenlin Biotechnology Co. Ltd (Jiangsu, China). They were housed under standard conditions (temperature: 18 -22 °C; relative humidity, 50% – 70%; noise level: 60 dB; 12 h light and dark cycle) and fed a standard rabbit diet and normal water *ad libitum*. The animal study was approved by the Institutional Ethics Committee of the Zhejiang Hospital. Corneal keratocytes were obtained from the rabbit eyes as previously described [48, 49]. Briefly, the corneal stroma layer was dissected into small fragments, digested with collagenase type II (Thermo Fisher Scientific, Waltham, MA, USA), and cultured in DMEM/F12 medium (Thermo Fisher Scientific) containing 10% FBS (Thermo Fisher Scientific) at 37°C for 7 days to generate myofibroblasts. The cellular phenotype of the myofibroblasts was monitored using a laser scanning confocal microscope (Olympus CX23 Tokyo, Japan).

### Immunofluorescence staining

Rabbit corneal keratocytes were characterized by immunofluorescence staining. Briefly, the keratocytes were permeabilized by incubating in 0.5% TritonX-10 for 20 min, and fixed with 4% paraformaldehyde for 20 min. Then, the cells were incubated overnight with primary antibodies, namely, anti-Vimentin (1:1000, Abcam Cambridge, MA, USA) and anti-cytokeratin K12 (CK12, 1:1000, Abcam) antibodies at 4°C. This was followed by incubation with the corresponding secondary antibodies (1:1000, Abcam) at 37°C for 1 h. Then, the cells were counterstained with the nuclear staining dye, DAPI, for 30 min, and photographed using a laser scanning confocal microscope (Olympus CX23 Tokyo, Japan).

### Western blotting

Total protein extracts were prepared by lysing the keratocytes and other cultured cells cells using the RIPA buffer (Beyotime, Shanghai, China) and the protein concentrations were measured using the BCA Protein Assay Kit (Thermo Fisher Scientific). Then, equal amounts of protein samples (30 μg per lane) were separated on 10 % SDS-PAGE gels, transferred onto PVDF membranes (Thermo Fisher Scientific), and blocked with 5% skimmed milk at room temperature. This was followed by incubation with primary antibodies against Keratocan (1:1000), Collagen I (1:1000), Collagen III (1:1000), MMP9 (1:1000), Fibronectin (1:1000), α-SMA (1:1000), GAPDH (1:1000), HIPK2 (1:1000), p-Smad3 (1:1000), Smad3 (1:1000), p-p53 (1:1000), p53 (1:1000) at 4°C overnight. Then, the membranes were incubated with the secondary goat anti-rabbit IgG antibody (1: 5000) at room temperature for 1 h. The blots were developed using ECL detection reagents (Thermo Fisher Scientific). The protein bands were scanned using the Odyssey infrared scanner (LICOR Biosciences, Lincoln, NE, USA), and analyzed with the Odyssey v2.0 software. All antibodies were obtained from Abcam.

### Isolation of rabbit ADCSs from adipose tissue

ADSCs were isolated from the subcutaneous adipose tissue obtained from the groin of the rabbits as previously described [50]. Briefly, the subcutaneous adipose tissue fragments were digested by incubation with collagenase type II and then treated with 10% FBS (Thermo Fisher Scientific) to terminate the enzyme reaction. The primary ADSCs were cultured for 15 days in DMEM/F12 medium containing 10% FBS and 100 U/mL streptomycin/penicillin at 37°C and 5% CO_2_.

### Flow cytometry

The primary ADSCs were stained with fluorescein-conjugated antibodies against CD29, CD90, CD34, and CD45 (Thermo Fisher Scientific) and analyzed using a BD flow cytometer (BD Biosciences, Mountain View, CA, USA). Briefly, the ADSCs were incubated on ice for 30 min with each antibody (1: 100 dilution), washed with brilliant stain buffer (BD Biosciences, Franklin Lake, NJ, USA), centrifuged to remove unbound antibodies in the supernatant, resuspended in brilliant stain buffer, and analyzed by flow cytometry.

### Isolation and characterization of rabbit ADSC exosomes

A total of 5 x 10^6^ ADSCs were grown in complete culture medium for 24 h. The medium was then replaced with serum-free DMEM/F12 medium and the cells were cultured for another 24 h. The exosomes were isolated from this cell culture medium using the Exosome isolation kit (Thermo Fisher Scientific) according to the manufacturer’s protocol. The ADSC exosomes (ADSCs-Exo) were pelleted by ultracentrifugation. The exosome pellet was resuspended in PBS and stored at -80°C. The ADSCs-Exos were characterized by nanoparticle tracking analysis (NTA), and western blotting. In the NTA assay, the size, distribution, and the number of particles in the ADSCs-Exo were evaluated using a nanoparticle tracking analyzer (v3.1, Malvern Instruments, Ltd., Worcestershire, UK). The ADSCs-Exo were analyzed by western blotting using the following primary antibodies: anti-CD9 (1:1000, Abcam), anti-CD81 (1:1000, Abcam), and anti-flotillin-1 (1:1000, Abcam).

### Cell proliferation assay

Cell proliferation was determined using the CCK-8 kit (Beyotime Biotechnology, Suzhou, China) according to the manufacturer's instructions. Briefly, rabbit corneal keratocytes were grown in DMEM/F12 medium containing 10% FBS at 37°C for 7 days. Then, 5 x 10^3^ rabbit corneal keratocytes per well were incubated for 0, 24, 48, 72 and 96 h at 37°C in the presence of 10% FBS. The control keratocytes were grown in serum-free DMEM/F12 medium. At the defined time points, cells were incubated with 10 μL of the CCK8 reagent at 37°C for another 2 h. Then, the optical density (OD) was determined at 450 nm using a microplate reader (Bio-Tek Instruments Inc., Winooski, VT, USA).

### HIPK2 kinase activity

Total protein lysates were prepared by cellular lysis using the RIPA buffer (Beyotime, Shanghai, China). HIPK2 activity in the samples was detected as previously described by Millipore (Calbiochem-Merck-Millipore, Darmstadt, Germany) [30]. Recombinant HIPK2 was used to construct a standard curve and the myelin basic protein (MBP) was used as the substrate.

### Quantitative reverse transcription PCR (qRT-PCR)

Total RNA from cell samples was extracted using the TRIzol reagent (Thermo Fisher Scientific) according to the manufacturer’s protocol. The cDNA synthesis was performed using the PrimeScript™ RT Master Mix Kit (Takara Biotechnology, Japan). The miRNAs were reverse transcribed into cDNA using specific reverse transcription (RT) primers with a MicroRNA Reverse Transcription Kit (HaiGene, Heilongjiang, China) according to the manufacturer’s specifications. The quantitative real time PCR (qRT-PCR) for mRNAs and miRNAs was performed using the SYBR Green™ Premix Ex Taq™ kit (Takara Biotechnology) in a Roche LightCycler® 96 (Roche, Basel, Switzerland). The concentration of specific mRNAs and miRNAs were determined using the 2^−ΔΔCt^ method, relative to GAPDH and U6, respectively. The qRT-PCR primers included the following: HIPK2: Forward, 5′-GATGCTGACCATAGATGCG-3′; Reverse, 5′-CATGGTCAAGTTGGTGGAT-3′. GAPDH: Forward, 5′-AGAGCACCAGAGGAGGACG-3′; Reverse, 5′-TGGGATGGAAACTGTGAAGAG-3′. Rabbit U6: 5′-TGCTACTTCTCACATGAAGACCT-3′. miR-19a-3p: 5′-TGCTCAAATCTATGCAAAACTGA-3′. miR-18a-5p: 5′-TGCTGTGCATCTAGTGCAGATAG-3′. miR-30c-5p: 5′-TGCTAACATCCTACACTCTCAGCT-3′.

### Cell transfections

The siRNAs targeting HIPK2 were purchased from GenePharma (Shanghai, China). Briefly, rabbit corneal keratocytes were grown in DMEM/F12 medium containing 10% FBS at 37°C for 7 days. Then, these cells were transfected with 5 μL siRNA for 6 h at 37°C according to the manufacturer’s protocol. Later, the medium was replaced with fresh DMEM/F12 medium containing 10% FBS and the cells were further incubated at 37°C for 42 h. QRT-PCR assay was used to estimate the levels of HIPK2 mRNA in the keratocytes. The sequences of the siRNAs used in this study were as follows: siRNA NC, sense: 5′-UUCUCCGAACGUGUCACGUTT-3′; anti-sense: 5′-ACGUGACACGUUCGGAGAATT-3′. HIPK2-siRNA1, sense: 5′-CAG AGAGUGCCGACGACUACA-3′; anti-sense: 5′-UAGUCGUCGGCACUCUCUGUG-3′. HIPK2-siRNA2, sense: 5′-AGACAACCAGGUUCUUCAACC-3′; anti-sense: 5′-UUGAAGAACCUGGUUCUCUUU-3′. HIPK2-siRNA3, sense: 5′-GGAAGGGAGCGACAUGUUAGU-3′; anti-sense: 5′-UAACAUGUCGCUCCCUUCCA -3′. Endogenous mature miR-19a agomir and agomir NC were purchased from GenePharma and transfected into ADSCs using Lipofectamine 2000 (Thermo Fisher Scientific) according to the manufacturer’s instructions. Fresh DMEM/F12 medium containing 10% FBS was added at 6 h after transfection, and the cells were further incubated at 37°C for 42 h. The levels of miR-19a were estimated using the qRT-PCR assay using the following primers: Agomir-NC, sense: 5′-UCACAACCUCCUAGAAAGAGUAGA-3′; anti-sense: 5′-UACUUUCUAGGAGGUUGUGAUU-3′. miR-19a agomir, sense: 5′-UGUGCAAAUCUAUGCAAAACUGA-3′; anti-sense: 5′-AGUUUUGCAUAGAUUUGCACAUU-3′.

### Lentivirus production and cell transfection

The pHBAd-CMV HIPK2 cDNA and lentiviral vector plasmids were obtained from GenePharma. The HIPK2 plasmids were co-transfected into 293T cells with the backbone plasmid (pHBAd-BHG). The lentiviral particles were collected from the supernatant at 72 h after transfection at 32°C and concentrated by centrifugation. The rabbit keratocytes (4 x 10^5^ cells / well) were grown in 60 mm cell plates at 37°C overnight. Then, the cells were transfected with HIPK2 cDNA-containing lentiviral supernatants for 24 h. The medium containing the virus was then replaced with fresh complete medium, and the positively transfected cells were selected in medium containing 2.5 μg/mL puromycin (Thermo Fisher Scientific) for 3 days. The qRT-PCR assay was used to assess the levels of HIPK2 in different experimental groups of keratocytes.

### Luciferase reporter assays

The HIPK2 3′UTR clone was purchased from GenePharma (Shanghai, China). The wild-type (WT) and mutant (MUT) HIPK2 3′-UTR’s were cloned into the PsiCHECK-2 vector. Then, 6 × 10^4^ 293T cells were seeded onto 48-well plates and co-transfected with PsiCHECK-2-HIPK2-WT or PsiCHECK-2-HIKP2-MUT constructs and miR-19a agomir using the Lipofectamine 2000 reagent (Thermo Fisher Scientific). After 48h, luciferase assay was performed using the dual luciferase reporter assay kit (Promega, Madison, WI, USA) according to the manufacturer’s instructions. The reporter luciferase activity was normalized to Renilla luciferase activity.

### Statistical analysis

All data were analyzed using the GraphPad Prism software version 7 for windows (GraphPad Software, La Jolla, CA, USA) and presented as mean ± SD. The differences between two experimental groups were analyzed using the Student’s t-test, whereas, comparisons between multiple experimental groups were estimated using the one-way analysis of variance (ANOVA) followed by Tukey’s test. All experiments were performed at least thrice and P < 0.05 was considered statistically significant.

## Supplementary Material

Supplementary Figures
