# Supplementary material for: Exosomal miR-19a from adipose-derived stem cells suppresses differentiation of corneal keratocytes into myofibroblasts
Source: Aging (Albany NY). 2020 Feb 29;12(5):4093–110. doi: 10.18632/aging.102802 (PMC7093196; doi:10.18632/aging.102802)
Supplement: Supplementary Figures [file aging-12-102802-s001..pdf]

SUPPLEMENTARY MATERIALS

Supplementary Figures

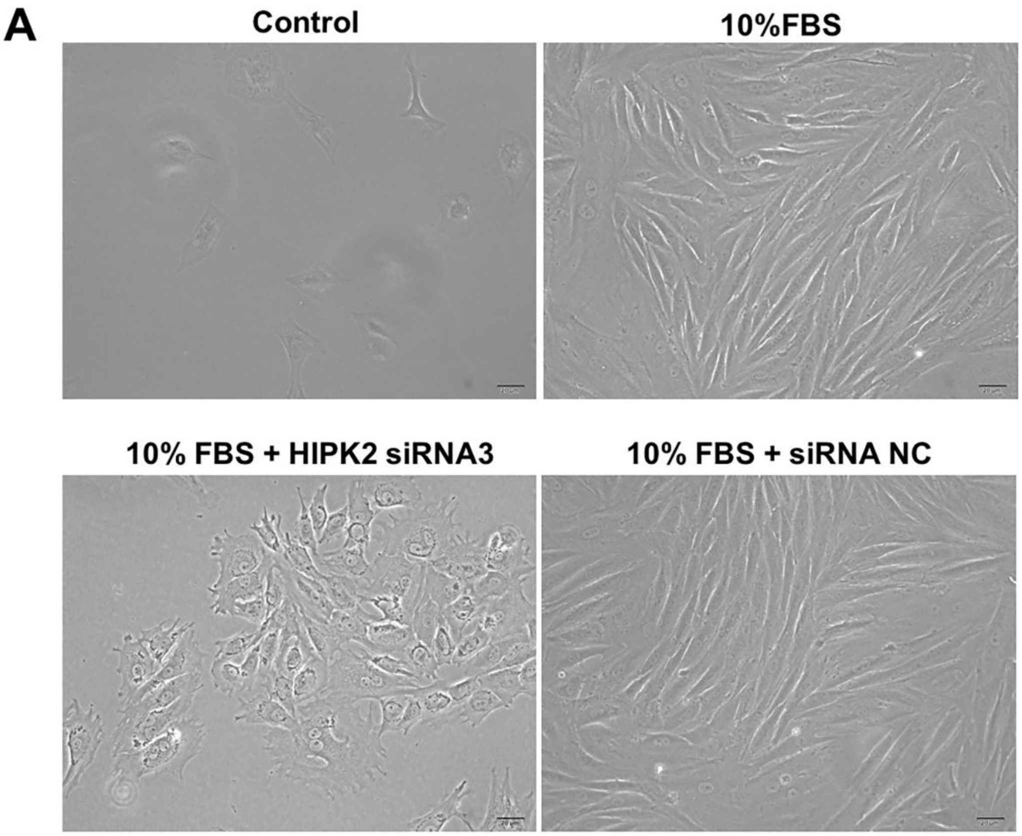

Supplementary Figure 1. Downregulation of HIPK2 prevents the myofibroblast phenotype promoted by FBS. Rabbit keratocytes were cultured in DMEM/F12 medium containing 10% FBS for 7 days. Then, cells were transfected with NC and HIPK2-siRNA3 in the presence of 10% FBS for another 48 h. (**A**) The cellular phenotype of cultures was observed using a microscope.

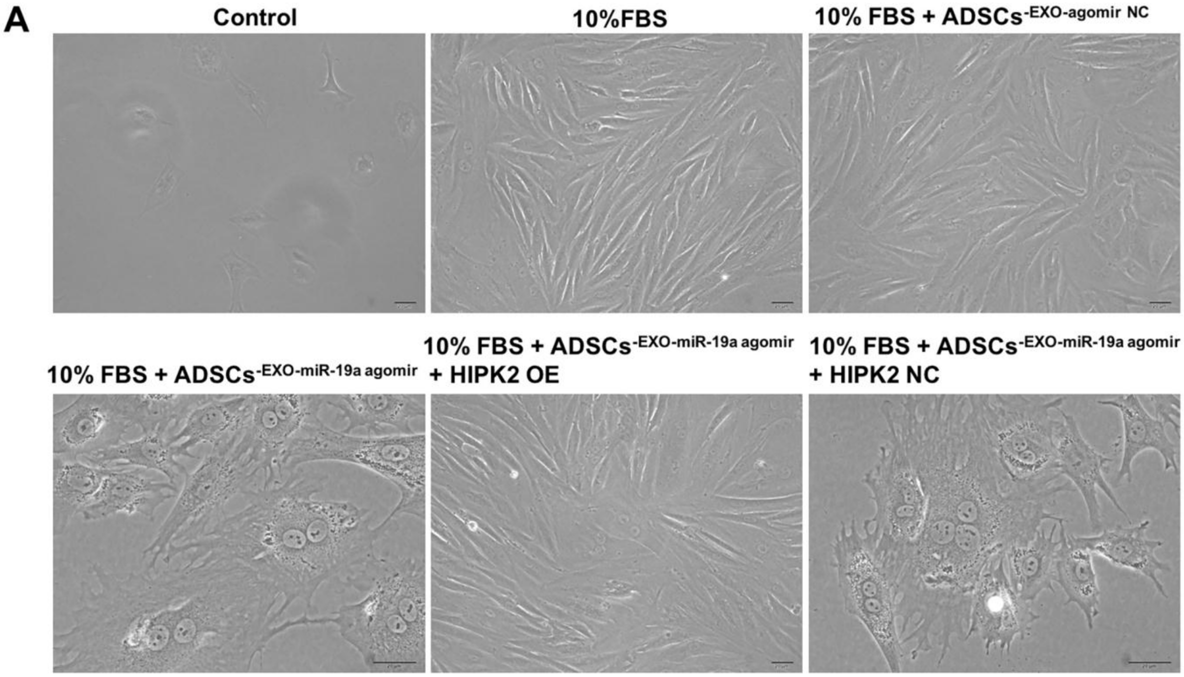

Supplementary Figure 2. ADSCs-Exo-miR-19a prevents the myofibroblast phenotype promoted by FBS. ADSCs were transfected with miR-19a agomir or agomir NC for 48 h, and then the pellet of ADSCs-Exo was collected. Meanwhile, keratocytes were cultured in DMEM/F12 medium containing 10% FBS for 7 days. Subsequently, cells incubated with 10% FBS were transfected with lenti-HIPK2 for 48 h in the presence of ADSCs-Exo (100 ug/mL). (**A**) The cellular phenotype of cultures was observed using a microscope.

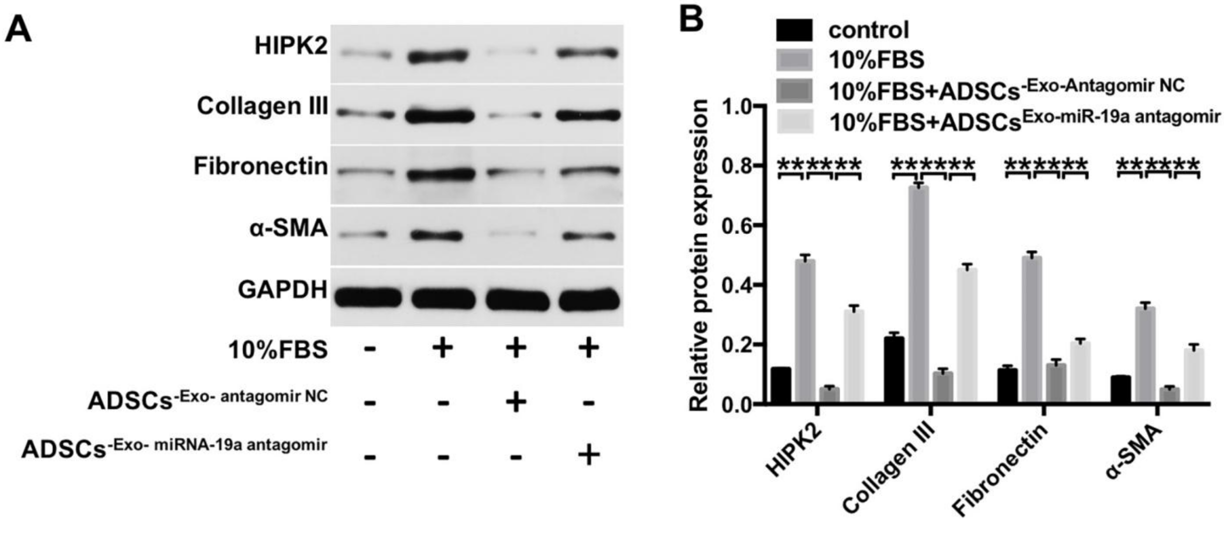

Supplementary Figure 3. Exosomes secreted from miR-19a knockdown ADSCs reverse the anti-fibrotic effects of ADSCs-Exo on the rabbit corneal keratocytes. (**A**, **B**) Western blot analysis shows levels of HIPK2, Collagen III, Fibronectin and α-SMA proteins in keratocytes grown in DMEM/F12 medium containing 10% FBS and treated with 100 μg/mL ADSCs-Exo-miR-19a-antagomir or 100 μg/mL ADSCs-Exo-NC-antagomir for 48 h. The ADSCs-Exo were obtained by ultracentrifugation of the cell culture medium in which ADSCs were grown after transfection with miR-19a-antagomir or NC-antagomir for 48 h. The expression of HIPK2, collagen III, fibronectin and α-SMA in cells were determined relative to GAPDH, which was used as an internal control. ** denotes $P < 0.01$.
